# Intermittent energy restriction changes the regional homogeneity of the obese human brain

**DOI:** 10.3389/fnins.2023.1201169

**Published:** 2023-08-03

**Authors:** Zhonglin Li, Xiaoling Wu, Hui Gao, Tianyuan Xiang, Jing Zhou, Zhi Zou, Li Tong, Bin Yan, Chi Zhang, Linyuan Wang, Wen Wang, Tingting Yang, Fengyun Li, Huimin Ma, Xiaojuan Zhao, Na Mi, Ziya Yu, Hao Li, Qiang Zeng, Yongli Li

**Affiliations:** ^1^Department of Radiology, Henan Provincial People’s Hospital, Zhengzhou University People’s Hospital, Henan University People’s Hospital, Zhengzhou, China; ^2^Department of Nuclear Medicine, Henan Provincial People’s Hospital, Zhengzhou University People’s Hospital, Henan University People’s Hospital, Zhengzhou, China; ^3^Henan Key Laboratory of Imaging and Intelligent Processing, PLA Strategic Support Force Information Engineering University, Zhengzhou, China; ^4^Health Mangement Institute, The Second Medical Center and National Clinical Research Center for Geriatric Diseases, Chinese PLA General Hospital, Beijing, China; ^5^Department of Nephrology, Henan Provincial Clinical Research Center for Kidney Disease, Henan Provincial Key Laboratory of Kidney Disease and Immunology, Henan Provincial People’s Hospital, Zhengzhou University People’s Hospital, Zhengzhou, China; ^6^Department of Nutrition, Henan Provincial People's Hospital, Zhengzhou University People's Hospital, Henan University People’s Hospital, Zhengzhou, China; ^7^Department of Health Management, Henan Key Laboratory of Chronic Disease Management, Henan Provincial People’s Hospital, Zhengzhou University People’s Hospital, Henan University People’s Hospital, Zhengzhou, China; ^8^Department of Oral Health Management, Fuwai Central China Cardiovascular Hospital, Zhengzhou, China

**Keywords:** intermittent energy restriction, regional homogeneity, obesity, weight loss, resting-state fMRI

## Abstract

**Background:**

Intermittent energy restriction (IER) is an effective weight loss strategy. However, the accompanying changes in spontaneous neural activity are unclear, and the relationship among anthropometric measurements, biochemical indicators, and adipokines remains ambiguous.

**Methods:**

Thirty-five obese adults were recruited and received a 2-month IER intervention. Data were collected from anthropometric measurements, blood samples, and resting-state functional magnetic resonance imaging at four time points. The regional homogeneity (ReHo) method was used to explore the effects of the IER intervention. The relationships between the ReHo values of altered brain regions and changes in anthropometric measurements, biochemical indicators, and adipokines (leptin and adiponectin) were analyzed.

**Results:**

Results showed that IER significantly improved anthropometric measurements, biochemical indicators, and adipokine levels in the successful weight loss group. The IER intervention for weight loss was associated with a significant increase in ReHo in the bilateral lingual gyrus, left calcarine, and left postcentral gyrus and a significant decrease in the right middle temporal gyrus and right cerebellum (VIII). Follow-up analyses showed that the increase in ReHo values in the right LG had a significant positive correlation with a reduction in Three-factor Eating Questionnaire (TFEQ)-disinhibition and a significant negative correlation with an increase in TFEQ-cognitive control. Furthermore, the increase in ReHo values in the left calcarine had a significant positive correlation with the reduction in TFEQ-disinhibition. However, no significant difference in ReHo was observed in the failed weight loss group.

**Conclusion:**

Our study provides objective evidence that the IER intervention reshaped the ReHo of some brain regions in obese individuals, accompanied with improved anthropometric measurements, biochemical indicators, and adipokines. These results illustrated that the IER intervention for weight loss may act by decreasing the motivational drive to eat, reducing reward responses to food cues, and repairing damaged food-related self-control processes. These findings enhance our understanding of the neurobiological basis of IER for weight loss in obesity.

## Introduction

Obesity is a complex chronic disease that has become a major public health problem in many countries throughout the world and contributes to the increased risk of many diseases, including cardiovascular disease, hypertension, hyperlipidemia, diabetes, and even certain cancers, thereby imposing huge social, medical, and economic burdens (Berthoud and Klein, 2017; Wang et al., 2022). Efforts to provide effective and economical strategies for weight loss in overweight or obese individuals are required to reduce the burden of obesity-related diseases (Wang et al., 2022).

Intermittent energy restriction (IER) is defined as periods of restricted energy intake followed by periods of normal energy intake (Wei et al., 2022). Recently, IER regimens have gained considerable popularity as an alternative to the orthodox continuous energy restriction (CER) approach for weight loss because many overweight or obese individuals have difficulty in maintaining the rigidity of CER (Schwingshackl et al., 2021; Wang et al., 2022; Wei et al., 2022). Furthermore, the effects of IER on weight loss are similar to those of CER (Schwingshackl et al., 2021; Wang et al., 2022; Wei et al., 2022). Existing studies have shown that IER can effectively reduce body weight and improve cardiometabolic outcomes in adolescents and adults with obesity (De Cabo and Mattson, 2019; Lister et al., 2020; Schwingshackl et al., 2021; Stanek et al., 2022; Wang et al., 2022; Wei et al., 2022). In July 2020, Wei et al. discovered that eight of nine short-term studies that used IER for weight loss showed improvement after treatment, and weight loss was observed to be sustained over the long term (Wei et al., 2022). Decreased leptin has also been found in an IER group compared with a control group after short-term intervention, indicating that IER alone is an effective intervention for improving vascular endothelial function (Bhutani et al., 2013). In addition, quality of life and eating behavior are improved, and self-esteem is increased (Lister et al., 2020; Schwingshackl et al., 2021; Stanek et al., 2022; Wei et al., 2022). Although IER interventions have been successful in curbing the obesity epidemic, the neurobiological bases that lead to beneficial dietary behavior changes and sustained weight loss are currently unclear.

Findings from brain neuroimaging studies have revealed that brain regions, including reward, cognitive control, emotional, and sensory circuits, contribute to the pathogenesis of obesity and weight management by regulating eating behavior (Carnell et al., 2012; Dagher, 2012; Ochner et al., 2013; Berthoud and Klein, 2017; Neseliler et al., 2019). Increased motivational drive to eat, increased reward responses to food cues, and impaired food-related self-control processes contribute to obesity (Berthoud et al., 2017). Compared with normal-weight individuals, obese individuals have greater activation of reward-related brain regions, such as the insula and orbitofrontal cortex (OFC), when processing food cues, and activation intensity is positively correlated with the calorie level of food cues (Pursey et al., 2014). However, reduced activation in the left dorsolateral prefrontal cortex (DLPFC) and insular cortex in response to food images has been observed in obese individuals (Brooks et al., 2013). The DLPFC and insular cortex are linked to cognitive control and interoceptive awareness, respectively, which may be associated with reduced bodily responses to the anticipation of food (such that great quantities of food need to be consumed to feel satisfied) and weakened attempts to control appetite (Brooks et al., 2013). These results indicate a weakened control system combined with hypersensitivity to satiety and discomfort signals after eating in persons who are prone to overeating (Brooks et al., 2013). Resting-state functional magnetic resonance imaging (fMRI) studies have revealed that obese individuals have lower functional connectivity (FC) in the middle frontal gyrus (a cortical region associated with attention, executive control, and movement) than normal-weight individuals (García-García et al., 2015). Parsons et al. (2022) concluded that altered FC of the OFC may indicate a shift in the valuation of food-based rewards, and dysfunctional insular FC likely contributes to altered homeostatic signal processing. Recently, Wang et al. revealed that connectomes within or between the visual cortex act as crucial neurobiological bases of obesity via machine learning and resting-state FC (Wang et al., 2023). Using methods, including the amplitude of low-frequency fluctuations (ALFFs) and the regional homogeneity (ReHo) of neural activity, some researchers have found changes in the spontaneous neural activity of obese brains, including brain regions implicated in reward (e.g., OFC and medial prefrontal cortex), emotion, and memory (e.g., amygdala and hippocampus) (Zhang et al., 2015, 2020).

Although neuroimaging studies on obesity have identified obesity-related abnormalities in a wide range of brain areas, these abnormalities can be reshaped after weight loss by CER involving reward cognitive control and sensory processing (Carnell et al., 2012; Zhang et al., 2015; Belaïch et al., 2017; Zeighami et al., 2021). Amanda et al. used the method of caloric restriction for weight loss in obese patients for 3 months and found that prefrontal cortical activation influences health behavior changes in the context of an intervention to produce weight loss and manage obesity (Szabo-Reed et al., 2020). After 6 months of calorie restriction, another study observed a significant correlation between the body mass index (BMI) change measured after 6 months and early alterations in fMRI food cue reactivity in the striatum, confirming that striatum reactivity to food cues is reshaped by diet (Hermann et al., 2019). Neseliler et al. (2019) reported that the activity of the DLPFC in the cognitive control circuit increases a month after calorie restriction and returns to baseline 3 months after calorie restriction; thus, it is positively correlated with weight loss. Moreover, weight loss intervention with very-low-calorie meals for 8 weeks decreases the blood oxygenation level-dependent signal in areas of the OFC and insula, so it is correlated with leptin levels and BMI (Van Opstal et al., 2019). These results indicated that obesity-associated alterations in neuronal activity are related to excessive body weight and may change after weight loss (Van Opstal et al., 2019). However, the weight loss interventions adopted by these studies were based on CER. The basis underlying chronological changes in brain activity induced by IER intervention remains unclear.

Recently, resting-state fMRI (rsfMRI) has been used to investigate functional alterations in obesity, and this method has unique advantages in clinical research (García-García et al., 2015; Zhang et al., 2015; Ding et al., 2020; Zhang et al., 2020; Parsons et al., 2022). ReHo is a data-driven method for rsfMRI that reflects spontaneous neuronal activity from different perspectives and demonstrates excellent performance in depicting clinical traits (Van Opstal et al., 2019; Zeighami et al., 2021; Li et al., 2022). It measures the similarity or synchronicity of the time series of the nearest neighboring voxels and can reflect the strength of local spontaneous neural activity in the brain (Zang et al., 2004; Li et al., 2022). This method has been successfully applied to reveal abnormalities in brain function in obese individuals. Obese men have significant differences in ReHo, including in the left putamen, OFC, and medial prefrontal cortex, relative to lean men before food intake, but this difference disappears after food intake (Zhang et al., 2015). Abnormal ReHo in the prefrontal cortex and precuneus has also been observed in obese individuals (Chao et al., 2018). Zhao et al. (2022) discovered that the ReHo of the right angular gyrus was smaller in obese undergraduates than in normal-weight undergraduates. These findings provide evidence of potential biomarkers for therapy and future research on obesity (Chao et al., 2018; Zhao et al., 2022). Gao et al. (2018) discovered that high impulsivity in food-related decision-making is inversely correlated with spontaneous ReHo in the dorsal caudate, proving that the dorsal striatum is one of the landmarks for overeating and weight change. Notably, decreased ReHo of the right DLPFC in young women mediates the association between baseline restrained eating and follow-up weight (Dong et al., 2015). Taken together, these findings revealed a possible underlying pathophysiology between restrained eating and the risk of weight gain (Dong et al., 2015). In addition, the ReHo method was used to study the effect of bariatric surgery-induced weight loss on brain activity (Zeighami et al., 2021). Zeighami et al. (2021) observed a significant increase in ReHo in the visual cortex, medial temporal gyrus, and DLPFC at 4 months after surgery. Therefore, ReHo analysis can be used to gain insights into the neural basis underlying IER for weight loss. However, findings regarding the changes in spontaneous neural activity after IER are lacking.

Moreover, the activity of brain circuits implicated in reward and self-regulation is modulated by internal states such as the current energy balance status (Neseliler et al., 2019; Hu et al., 2021). During calorie restriction, leptin reflects changes in energy homeostasis, and its levels decline rapidly and then slowly with the reduction in fat mass (Friedman and Mantzoros, 2015). Cross-talk between adipose tissue and the central nervous system underlies the increased risk of developing brain diseases such as cognitive and mood disorders in obese people (Forny-Germano et al., 2019). Leptin and adiponectin are two of the most abundant and well-studied adipokines in the brain, with particular emphasis on how the altered signaling of these adipokines in obesity may lead to cognitive dysfunction (Forny-Germano et al., 2019). Leptin is an adipokine secreted predominantly by white adipose tissue and provides feedback to the hypothalamus regarding peripheral fat stores (Forny-Germano et al., 2019). Reduced leptin levels have been observed after IER interventions (Harvie et al., 2011; Pinto et al., 2020). Previous studies have suggested that reductions in leptin levels can result in increased activity in the mesolimbic reward system and, possibly, reduced activity in brain regions associated with cognitive control (Neseliler et al., 2019; Zeighami et al., 2021). Leticia et al. indicated that an improved understanding of the role of leptin in brain disorders may be relevant for diagnosis, prevention, and therapy (Forny-Germano et al., 2019). However, the interactions between spontaneous neural activity and adipokines (leptin and adiponectin) during IER interventions in obesity remain unclear.

In summary, obesity is associated with abnormal brain function, and IER is effective in reducing excessive body weight. However, the accompanying changes in spontaneous neural activity remain unclear, and the relationship among anthropometric measurements, biochemical indicators, and adipokines is ambiguous. Here, we hypothesized that the IER intervention induces significant changes in spontaneous neural activity in brain regions related to reward, appetite control, and visual attention, and these improvements are associated with changes in anthropometric measurements, biochemical indicators, and adipokines. To test this hypothesis, we used rsfMRI to characterize dynamic changes in spontaneous neural activity (ReHo) during 2 months of the IER intervention in obese individuals. We also investigated the relationships between the ReHo values of the altered regions and changes in anthropometric measurements, biochemical indicators, and adipokines (leptin and adiponectin).

## Methods

### Participants

This study was approved by the Ethics Committee of Henan Provincial People’s Hospital [ethical approval number: 2018(18)] and registered at https://clinicaltrials.gov (Protocol ID: 20180520). All procedures were performed according to the relevant ethical regulations, Helsinki Declaration, and privacy legislation. All the participants provided written informed consent. The inclusion criteria were as follows: (1) women or men who met the World Health Organization Guidelines for overweight in Asian populations, that is, BMI ≥ 27.5 kg/m^2^, and (2) age between 18 and 60 years. Moreover, all participants had well-controlled hypertension, diabetes, hyperlipidemia, hyperuricemia, or metabolic syndrome. The exclusion criteria were as follows: (1) serious cardiopathy, (2) liver dysfunction, (3) renal disease, (4) hypoglycemia, (5) anemia, (6) malnutrition, (7) hematopoietic system disease, (8) systemic immune system disease, (9) infectious disease, (10) pregnancy, (11) neurological disorders, and (12) contraindications to MRI. A total of 35 adults with obesity (BMI 35.29 ± 4.15 kg/m^2^; age 36.26 ± 9.14 years; 16 females) were recruited from hospitals from April to November 2018. The recruitment process is illustrated in Supplementary Figure S1. To explore the neuroimaging bases for successful weight loss and prevent the data of failed weight loss subjects from interfering with the experimental results, in accordance with the clinical practice guidelines for the medical care of individuals with obesity in America published by Garvey et al. (2016), we classified subjects whose weight loss was greater than 5% into the successful weight loss group (SWLG) in this study, and those whose weight loss was less than 5% were defined as the failed weight loss group (FWLG). Ultimately, 25 obese individuals were included in the SWLG, and five obese individuals were included in the FWLG for further analysis.

### IER protocol

**Figure 1:**
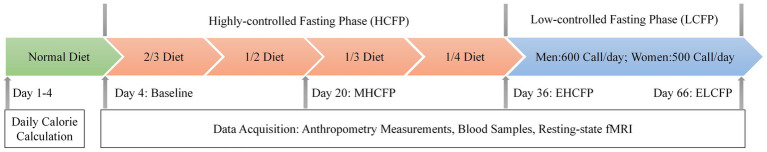
Procedure of the IER intervention experiment. The average daily calorie intake of each participant was calculated based on a normal diet. Anthropometry measurements, blood samples, and resting-state fMRI data were collected before HCFP (day 4: baseline), at the midpoint of HCFP (day 20: MHCFP), at the endpoint of HCFP (day 36: EHCFP), and at the endpoint of LCFP (day 66: ELCFP). MHCFP, midpoint of the highly controlled fasting phase; EHCFP, endpoint of the highly controlled fasting phase; ELCFP, endpoint of the low-control fasting phase.

The IER protocol is illustrated in Figure 1. The intervention design and caloric intake criteria were based on the studies by Wei et al. (2017) and Zeng et al. (2021). In the normal diet phase, the participants were on a normal diet without restrictions in calories and food types for 4 days. The average daily calorie intake of each participant was calculated based on 24-h dietary recalls and 4 days of food records, which is a feasible means of collecting dietary data for nutrition research (Park et al., 2018). This study was conducted by a nutritionist at the hospital. The highly controlled fasting phase (HCFP) contained four periods (a total of 32 days), each of which lasted for 8 days. Each participant was on a diet with 2/3 (period 1), 1/2 (period 2), 1/3 (period 3), and 1/4 (period 4) of the average daily caloric intake in the normal-diet phase every other day. The participants received multivitamin intervention (3 g/day) in periods 1 and 2. The participants received dietary fiber (15 g/day) and multivitamin intervention (3 g/day) in periods 3 and 4. Daily dietary calories included 45% carbohydrates, 45% fat, and 10% protein. Specific food information for each stage is presented in Supplementary Table S3. In the low-control fasting phase (LCFP) lasting for 30 days, participants were provided a calorie-restricted diet (less than 600 calories/day for men and less than 500 calories/day for women) every other day. Anthropometric measurements, blood samples, and rsfMRI data were collected before HCFP (day 4: baseline), at the midpoint of HCFP (day 20: MHCFP), at the endpoint of HCFP (day 36: EHCFP), and at the endpoint of LCFP (day 66: ELCFP), respectively.

### Anthropometric measurement

Body weight (BW), BMI, waist circumference (WC), body fat (BF), percentage of body fat (PBF), skeletal muscle (SM), systolic blood pressure (SBP), and diastolic blood pressure (DBP) were measured at baseline, MHCFP, EHCFP, and ELCFP. The subjects were required to complete a 21-item Chinese version of the Three-Factor Eating Questionnaire (TFEQ), which assesses three domains of eating behavior: disinhibition, cognitive control, and hunger (Stunkard and Messick, 1985). High scores indicated uncontrolled eating, cognitive restraint, and emotional eating.

### Blood sample collection and analysis

Blood samples were collected in the morning (7:00–8:00) after overnight fasting at the different time points mentioned above. Serum was obtained by centrifugation at 3,000 rpm for 15 min at 23°C and stored at −80°C until use. The serum levels of fasting plasma glucose (FPG), glycosylated hemoglobin (HbA1c), total cholesterol (TC), triglyceride (TG), high-density lipoprotein (HDL), low-density lipoprotein (LDL), aspartate transaminase (AST), alanine aminotransferase (ALT), glutamyl transpeptidase (GGT), alkaline phosphatase (ALP), serum creatinine (SCR), and uric acid (UA) were measured in the clinical laboratory of our hospital. Leptin and adiponectin levels were measured using ELISA (Thermo Multiskan MK3, USA).

### MRI acquisition and analysis

A MAGNETOM Prisma 3 T MR scanner (Siemens Healthcare, Erlangen, Germany) with a 64-channel head–neck coil was used for fMRI data acquisition at the Medical Imaging Center of our hospital. Earplugs and foam pads were used to minimize scanner noise and head motion. Medical tape was fixed on the participants’ foreheads to help them control their movements. rsfMRI data were acquired between 7 and 10 p.m. using an echo-planar imaging sequence with 200 volumes lasting for 400 s. All participants were asked to keep their eyes open and fixate on a green cross in the middle of the screen during rsfMRI scanning. The corresponding acquisition parameters were set as follows: repetition time (TR), 2,000 ms; echo time (TE), 35 ms; field of view (FOV), 220 mm × 220 mm; matrix size, 94 × 94; slices, 75; slice thickness, 2.2 mm; flip angle (FA), 80°; and SMS factor, 3. High-resolution T1-weighted structural images were acquired using the following parameters: TR, 2,300 ms; TE, 2.27 ms; FOV, 250 mm × 250 mm; matrix size, 256 × 256; slices, 192; slice thickness, 1 mm; and FA, 8%.

Data preprocessing and ReHo analysis were performed using the Data Processing and Analysis of Brain Imaging toolbox (Yan et al., 2016).^1^ First, we discarded the first 10 volumes of each run for signal stabilization and participant adaptation. Second, slice timing and head motion corrections were conducted. Data with a maximum displacement in head rotation greater than 2° or any direction greater than 2 mm were excluded from further analysis. Third, for precise spatial normalization of the fMRI data, individual high-resolution T1-anatomic images were registered to the mean fMRI data, and the resulting aligned T1-weighted images were segmented and transformed into standard Montreal Neurological Institute space using the DARTEL toolbox. Fourth, white matter, cerebrospinal fluid signals, and 24 head realignment parameters were regressed as covariates. The regressed functional images were normalized to the group template by using the transfer parameter estimated by DARTEL segmentation and resampled to 3 mm^3^ × mm^3^ 3 × 3 mm^3^ voxels. Lastly, a linear trend and temporal bandpass filtering (0.01–0.1 Hz) were applied to reduce low-frequency drift and physiological high-frequency respiratory and cardiac noise.

ReHo maps were generated for the preprocessed rsfMRI data as previously described (Dong et al., 2015; Zhang et al., 2015; Chao et al., 2018; Gao et al., 2018; Zeighami et al., 2021; Zhao et al., 2022). Kendall’s coefficient of concordance (KCC) was calculated to measure the similarity between the time series of a given voxel and those of its nearest 26 voxels. To reduce the influence of individual variations in KCC values, we performed ReHo map normalization by dividing the KCC of each voxel by the averaged whole-brain KCC. The ReHo maps were spatially smoothed using a Gaussian kernel with a 6 mm full-width at half-maximum. To assess the time effects on the obese brain, we performed repeated-measures ANOVA to compare the ReHo maps of the SWLG and FWLG at baseline, MHCFP, EHCFP, and ELCFP during the IER intervention, followed by Bonferroni-corrected post-hoc paired t-tests. To analyze the correlation of signals from brain regions that exhibited altered ReHo across the four time points, we extracted signals from these brain regions by using the Anatomical Automatic Labeling template.

### Statistical analysis

All statistical analyses were performed using Statistical Product and Service Solutions version 26.0 (IBM Corporation). Repeated-measures ANOVA was applied to compare the anthropometric measurements, biochemical indicators, and ReHo of the brain activity of the participants at baseline, MHCFP, EHCFP, and ELCFP during IER (Hu et al., 2021). Bonferroni post-hoc pairwise comparisons were conducted to examine differences in factor means when significant *F* values were obtained during ANOVA. Given that repeated-measures ANOVA was conducted within the same group and the time points as the experimental factor, no covariate was used for this analysis, as in studies by Hu et al. (2021) and Zeighami et al. (2021). The distribution of anthropometric measurements and biochemical indicators was tested using the Shapiro–Wilk method. Continuous variables were expressed as the mean ± standard deviation. The threshold for statistical significance was set at *p* < 0.05.

### Correlation analysis

To identify possible associations between the ReHo of altered brain regions and anthropometric measurements, we applied significant changes in corresponding values from the baseline to ELCFP after the IER intervention to partial (normally distributed data) or Spearman (non-normally distributed data) correlation analyses with age and sex as covariates, together with the adipokines (leptin and adiponectin) and biochemical indicators (Neseliler et al., 2019). All reported *p*-values were adjusted for multiple comparisons using the false discovery rate method. Statistical significance was set at *p* < 0.05.

## Results

### Effect of IER on anthropometric measurements

**Figure 2:**
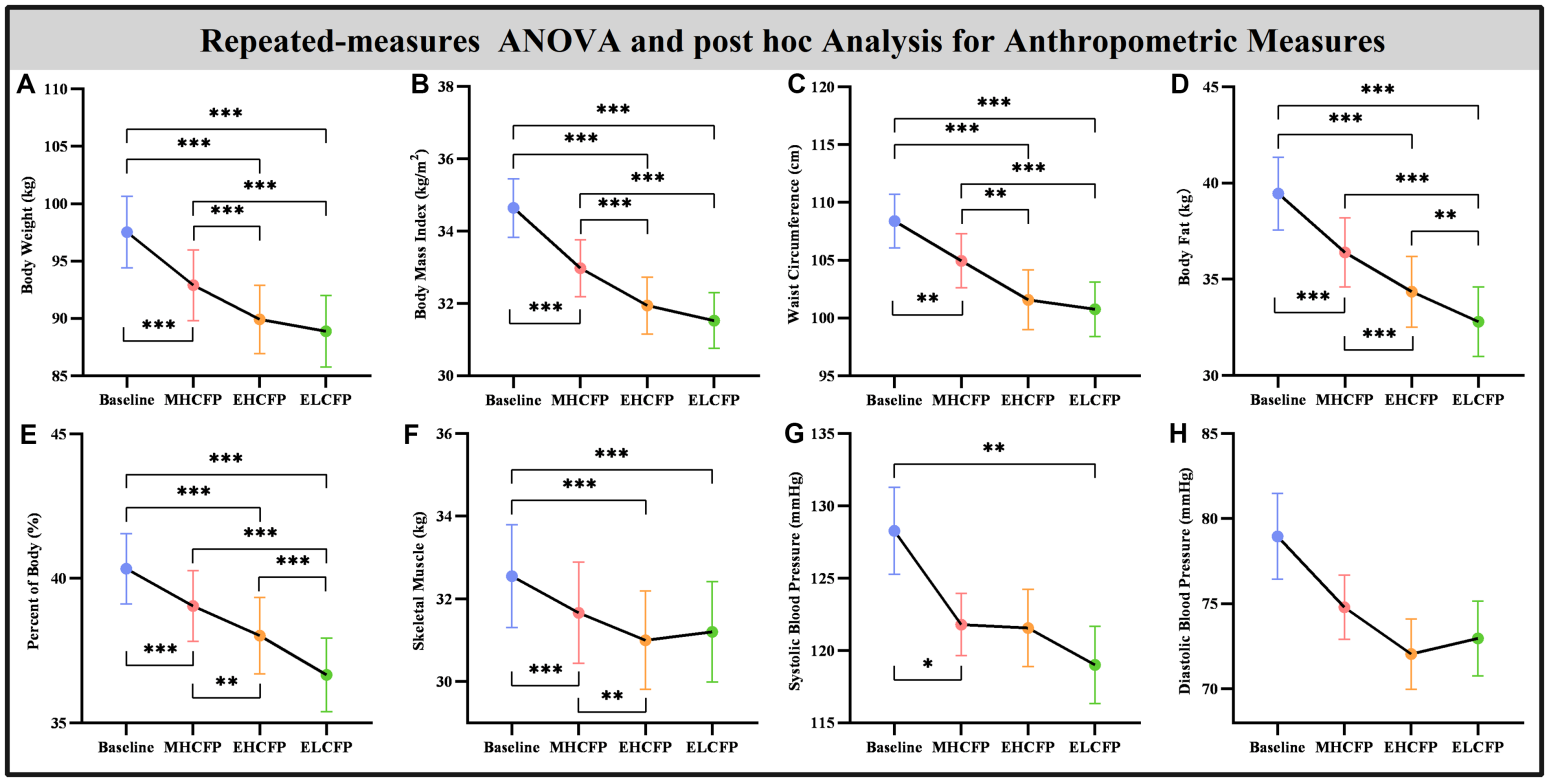
Effect of IER on anthropometry measurements. Repeated-measures ANOVA indicated that the IER intervention resulted in a significant reduction in all anthropometric measurements, including body weight **(A)**, body mass index **(B)**, waist circumference **(C)**, body fat **(D)**, percent of body fat **(E)**, skeletal muscle **(F)**, systolic blood pressure **(G)**, and diastolic blood pressure **(H)**, across the four time points. Data are expressed as the mean ± standard error of the mean. Abbreviations: endpoint of the highly controlled fasting phase, EHCFP; endpoint of the low-control fasting phase, ELCFP. *p < 0.05, **p < 0.01, ***p < 0.001.

**Figure 3:**
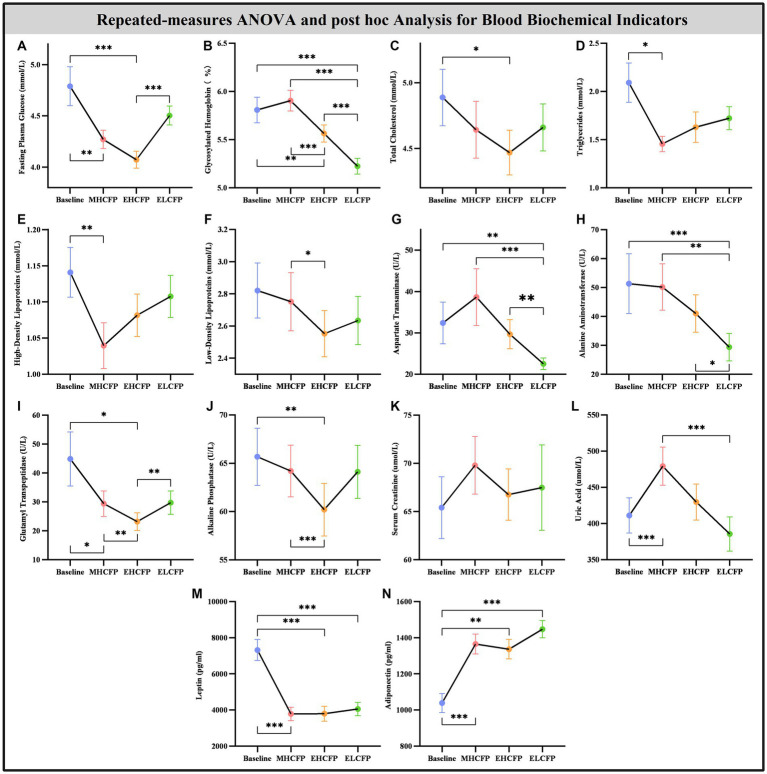
Effect of intermittent energy restriction on blood biochemical indicators. Repeated-measures ANOVA indicated that the IER intervention resulted in significant changes in blood biochemical indicators, including fasting plasma glucose **(A)**, glycosylated hemoglobin **(B)**, total cholesterol **(C)**, triglycerides **(D)**, high-density lipoproteins **(E)**, low-density lipoproteins **(F)**, aspartate transaminase **(G)**, alanine aminotransferase **(H)**, glutamyl transpeptidase **(I)**, alkaline phosphatase **(J)**, serum creatinine **(K)**, uric acid **(L)**, leptin **(M)**, and adiponectin **(N)** (except for serum creatinine), across the four time points. Data are expressed as the mean ± standard error of the mean. Abbreviations: midpoint of the highly controlled fasting phase, MHCFP; endpoint of the highly controlled fasting phase, EHCFP; endpoint of the low-control fasting phase, ELCFP. *p < 0.05, **p < 0.01, ***p < 0.001.

The demographic data of the SWLG were as follows: 12 females aged 37.64 ± 9.37 years. All anthropometric measurements were normally distributed. Figure 2 and Supplementary Table S1 present the results of anthropometric measurements obtained using repeated-measures ANOVA and post-hoc comparisons. Repeated-measures ANOVA indicated that the IER intervention resulted in all anthropometric measurements exhibiting a significant reduction across the four time points, namely, BW [*F*(3,72) = 117.54, *p* < 0.001], BMI [*F*(3,72) = 103.78, *p* < 0.001], WC [*F*(3,69) = 29.85, *p* < 0.001, missing data for one subject], BF [*F*(3,72) = 126.41, *p* < 0.001], PBF [*F*(3,72) = 64.07, *p* < 0.001], SM [*F*(3,72) = 27.53, *p* < 0.001], SBP [*F*(3,72) = 5.635, *p* = 0.002], and DBP [*F*(3,72) = 4.324, *p* = 0.007]. Post-hoc comparisons (ELCFP vs. baseline) indicated that anthropometric measurements decreased significantly (all *p ≤* 0.001) after the end of the IER intervention, except for DBP. Figure 3 and Supplementary Table S1 also present the results of the TFEQ obtained by repeated-measures ANOVA and post-hoc comparisons. The data of two subjects were missing. The results indicated that the IER intervention resulted in a significant reduction in TFEQ-disinhibition [*F*(3,66) = 8.3757, *p* < 0.001] and a significant increase in TFEQ-cognitive control [*F*(3,66) = 9.984, *p* < 0.001] across the four time points, except for TFEQ-hunger [*F*(3,66) = 1.884, *p* = 0.141]. The Cronbach’s alphas of TFEQ-disinhibition, TFEQ-cognitive control, and TFEQ-hunger were 0.742, 0.665, and 0.756, respectively. The Cronbach’s alpha of the entire TFEQ was 0.787. The demographic data of the FWLG were as follows: two females aged 37.40 ± 8.20 years. Supplementary Table S1 presents the detailed results of the anthropometric measurements in the FWLG.

### Effect of IER on biochemical indicators

**Figure 4:**
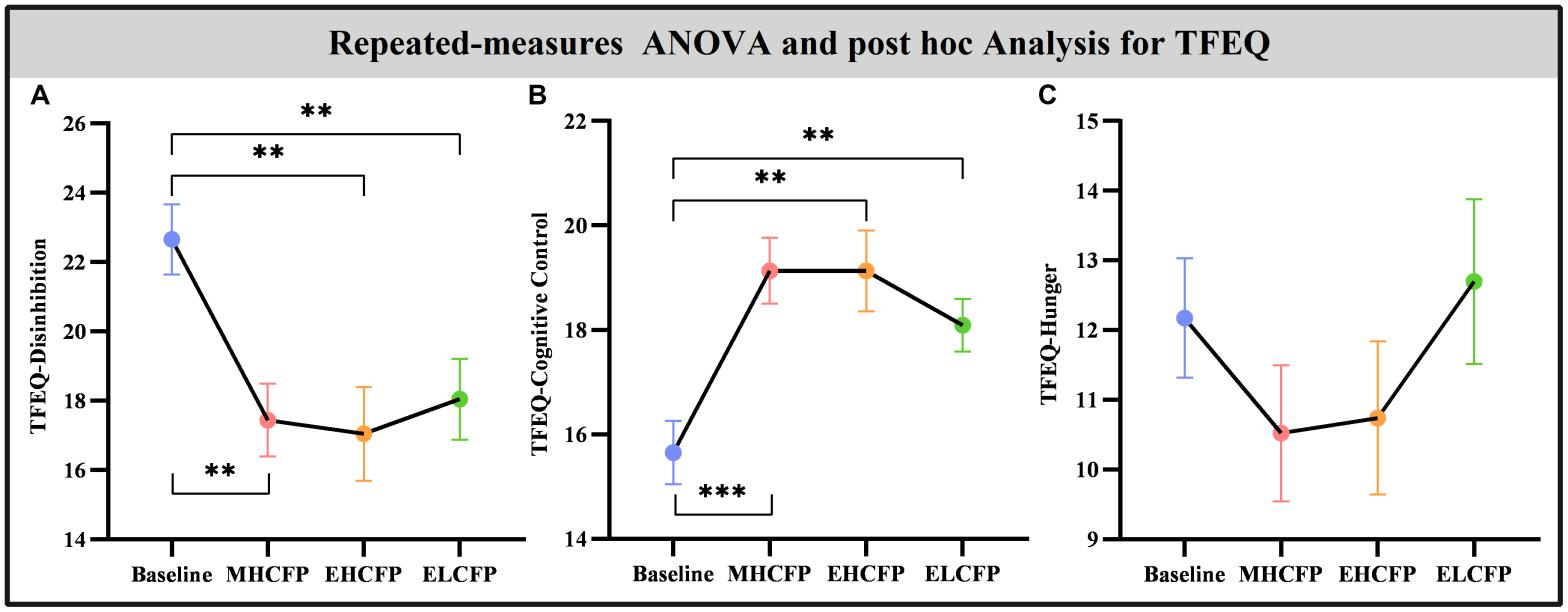
Effect of intermittent energy restriction on TFEQ. Repeated-measures ANOVA indicated that the IER intervention resulted in a significant reduction in TFEQ-disinhibition **(A)** and a significant increase in TFEQ-cognitive control **(B)** across the four time points, except for TFEQ-hunger **(C)**. Data are expressed as the mean ± standard error of the mean. Abbreviations: Three-factor Eating Questionnaire, TFEQ; midpoint of the highly controlled fasting phase, MHCFP; endpoint of the highly controlled fasting phase, EHCFP; endpoint of the low-control fasting phase, ELCFP. **p < 0.01 and ***p < 0.001.

Figure 4 and Supplementary Table S2 show the results of the biochemical indicators and adipokines (leptin and adiponectin) obtained by repeated-measures ANOVA and post-hoc comparisons. Repeated-measures ANOVA indicated that the IER intervention resulted in significant changes in some blood biochemical indicators across the four time points, including FPG [*F*(3,72) = 14.455, *p* < 0.001], HbA1c [*F*(3,72) = 71.239, *p* < 0.001], TC [*F*(3,72) = 3.788, *p* = 0.022], TG [*F*(3,72) = 3.835, *p* = 0.013], HDL [*F*(3,72) = 4.936, *p* = 0.004], LDL [*F*(3,72) = 3.587, *p* = 0.018], AST [*F*(3,72) = 14.203, *p* < 0.001], ALT [*F*(3,72) = 13.267, *p* < 0.001], GGT [*F*(3,72) = 8.718, *p* = 0.005], ALP [*F*(3,72) = 6.513, *p* = 0.002], UA [*F*(3,72) = 13.955, *p* < 0.001], leptin [*F*(3,72) = 21.586, *p* < 0.001], and adiponectin [*F*(3,72) = 15.334, *p* < 0.001]. However, the IER intervention had no significant effect on SCR [*F*(3,72) = 2.357, *p* = 0.118]. Post-hoc comparisons (ELCFP vs. baseline) indicated that several blood biochemical indicators decreased significantly after the end of the IER intervention, including HbA1c, AST, ALT, leptin, and adiponectin (all *p* < 0.01, Figure 4; Supplementary Table S2). Supplementary Table S2 presents the detailed results of biochemical indicators and adipokines in the FWLG.

### Effect of IER on ReHo

**Figure 5:**
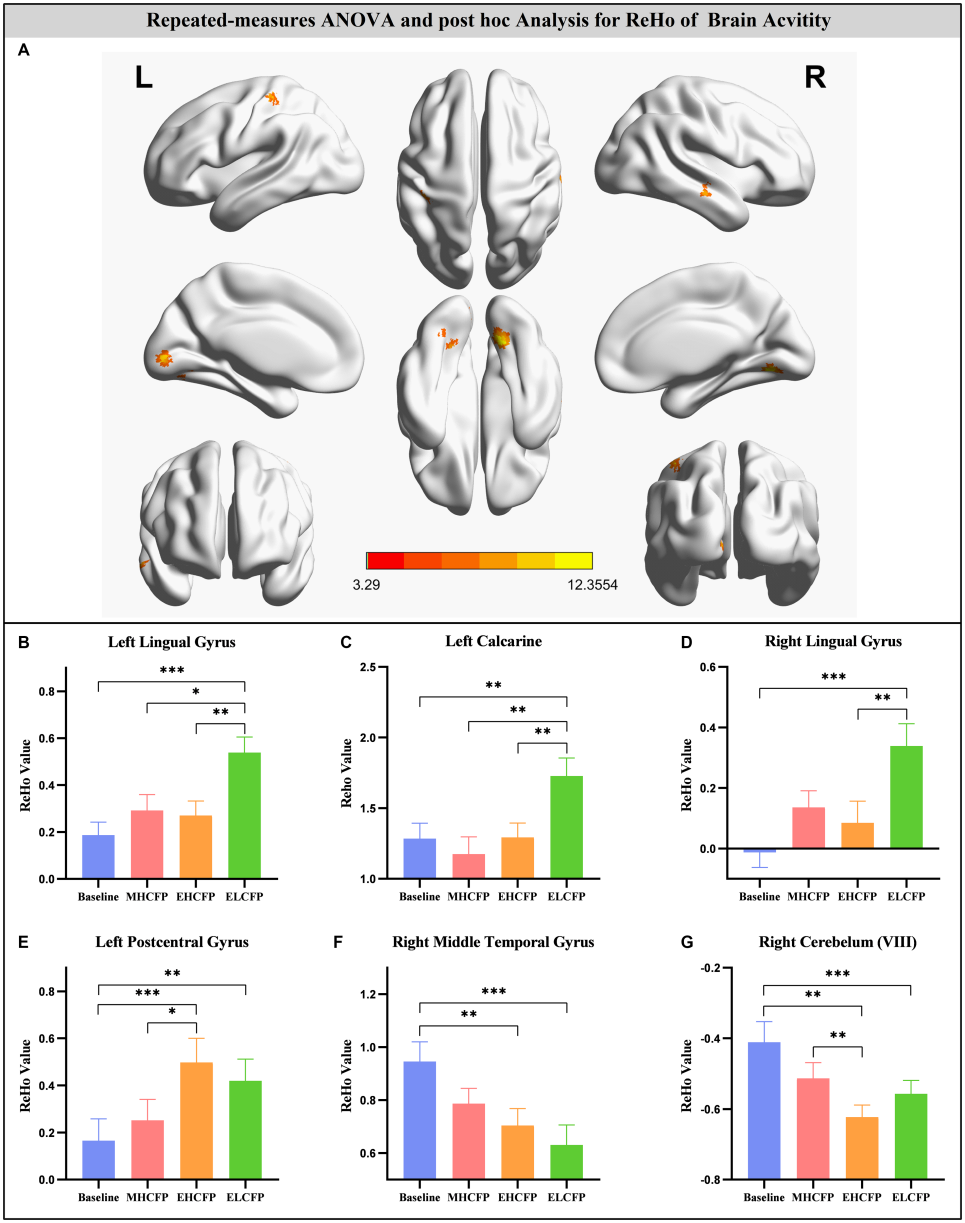
Brain regions exhibited changed ReHo in individuals with obesity induced by intermittent energy restriction intervention **(A)**. Repeated-measures ANOVA indicated that the IER intervention resulted in a significant increase in ReHo in the bilateral lingual gyrus **(B,D)**, left calcarine **(C)**, and left postcentral gyrus **(E)** and a significant decrease in the right middle temporal gyrus **(F)** and right cerebellum (VIII) **(G)**. Data are expressed as the mean ± standard error of the mean. Abbreviations: regional homogeneity, ReHo; midpoint of the highly controlled fasting phase, MHCFP; endpoint of the highly controlled fasting phase, EHCFP; endpoint of the low-control fasting phase, ELCFP; L, left; R, right. *p < 0.05, **p < 0.01, ***p < 0.001.

For the SWLG, repeated-measures ANOVA indicated a significant increase in ReHo in the bilateral lingual gyrus (LG), left calcarine, and left postcentral gyrus (PCG) and a significant decrease in the right middle temporal gyrus (MTG) and right cerebellum (VIII). Table 1 provides detailed information on the activity centers. The spatial distribution of the altered ReHo regions is shown in Figure 5A. The results were set at the voxel level (*p* < 0.001) and cluster level (*p* < 0.05) (*t* = 3.29, GRF-corrected). The post-hoc comparisons and distribution of z-transformed ReHo values in the brain regions that exhibited altered ReHo across the four time points are shown in Figures 5B–G. However, no significant difference in the FWLG was observed at the voxel (*p* < 0.001) and cluster levels (*p* < 0.05) (*t* = 3.29, GRF corrected).

**Table 1:** Brain regions exhibited changed ReHo in patients with obesity induced by intermittent energy restriction intervention.

Brain regions	Whole cluster size	Cluster size	MNI coordinates			*F* score
			*x*	*y*	*z*	
Left lingual gyrus	76	33	−12	−81	0	8.93
Left calcarine		31	−9	−84	0	10.32
Right lingual gyrus	61	55	15	−66	−6	12.20
Left postcentral gyrus	51	47	−45	−33	60	12.36
Right middle temporal gyrus	44	16	60	−18	−12	9.01
Right cerebellum (VIII)	39	37	30	−57	−48	11.48

The results were set at the voxel level: *p* < 0.001, cluster-level: *p* < 0.05, *t* = 3.29 (Gaussian random field-corrected). ReHo, Regional homogeneity; MNI, Montreal Neurological Institute.

### Associations between changes in ReHo signals and anthropometric measurements/biochemical indicators/adipokines after IER for SWLG

**Figure 6:**
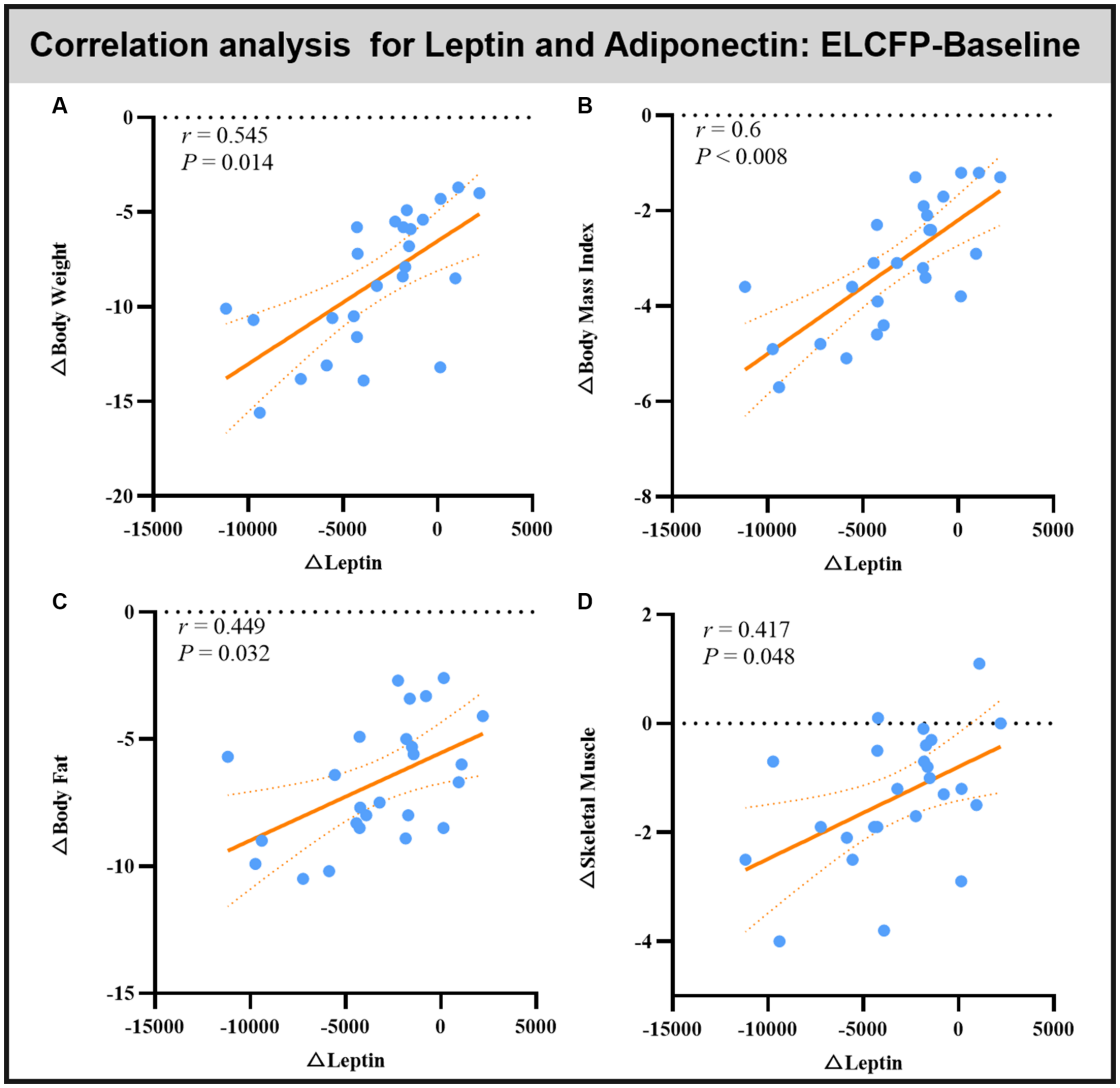
Scatter plots showing the relationship between changes in leptin/adipokines and changes in body weight **(A)**, body mass index **(B)**, body fat **(C)**, and skeletal muscle **(D)** for the comparison of ELCFP with the baseline during IER. Abbreviation: endpoint of the low-control fasting phase, ELCFP.

**Figure 7:**
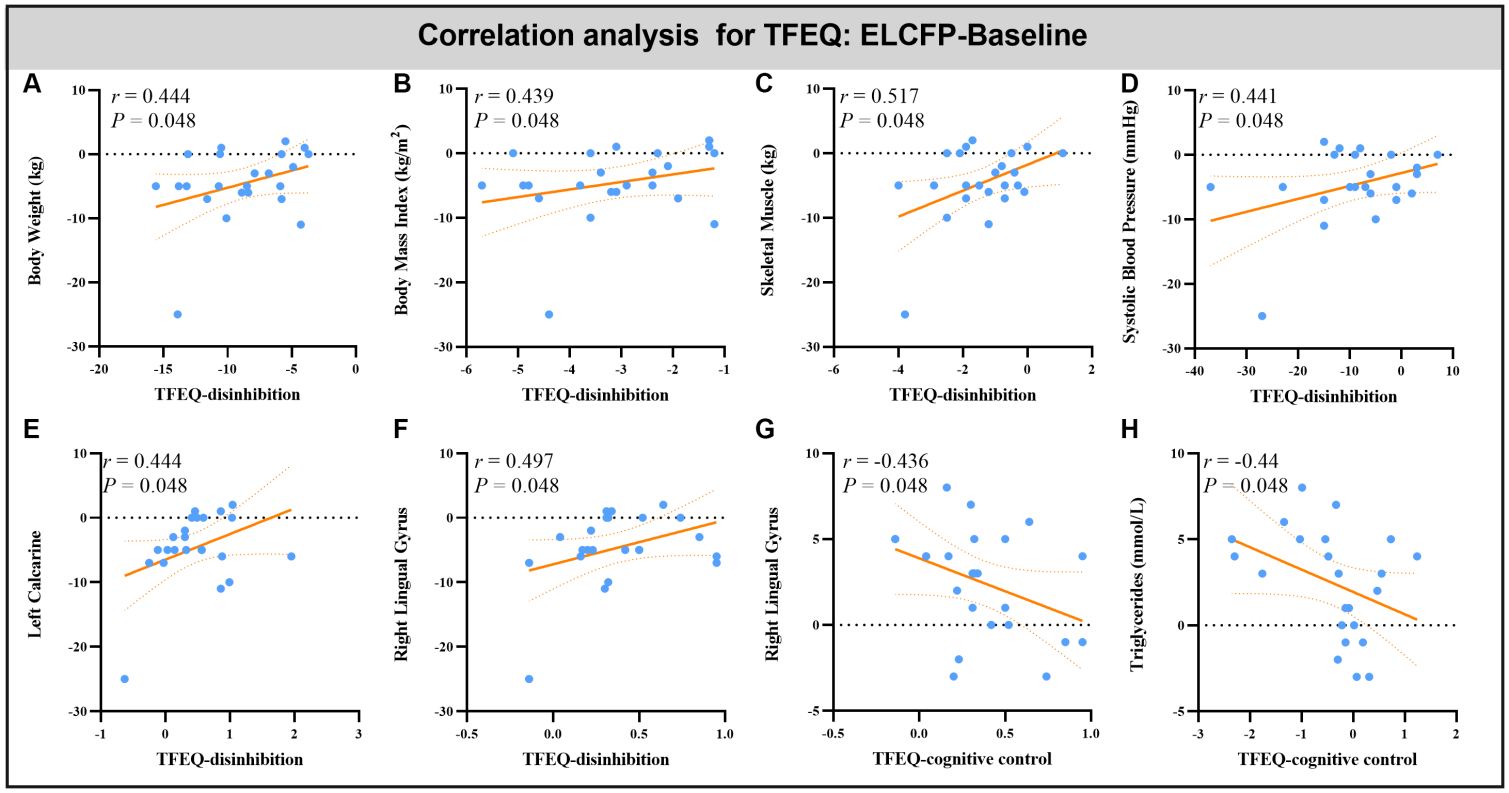
Scatter plots showing the relationship between changes in TFEQ and changes in body weight **(A)**, body mass index **(B)**, skeletal muscle **(C)**, systolic blood pressure **(D)**, triglyceride **(H)** and ReHo values in altered brain regions [left calcarine **(E)** and right lingual gyrus **(F,G)**] for the comparison of ELCFP with the baseline during IER. Abbreviations: regional homogeneity, ReHo; Three-factor Eating Questionnaire, TFEQ; endpoint of the low-control fasting phase, ELCFP.

Leptin reduction was positively correlated with BW (*r* = 0.545, *p* = 0.014, Figure 6A), BMI (*r* = 0.6, *p* < 0.008, Figure 6B), BF (*r* = 0.449, *p* = 0.032, Figure 6C), and SM (*r* = 0.483, *p* = 0.018, Figure 6D). The reduction in TFEQ-disinhibition showed a significant positive correlation with changes in BW (*r* = 0.444, *p* = 0.048, Figure 7A), BMI (*r* = 0.439, *p* = 0.048, Figure 7B), SM (*r* = 0.517, *p* = 0.048, Figure 7C), SBP (*r* = 0.441, *p* = 0.048, Figure 7D), ReHo of the left calcarine (*r* = 0.444, *p* = 0.048, Figure 7E), and ReHo of the right LG (*r* = 0.497, *p* = 0.048, Figure 7F). The increase in TFEQ-cognitive control showed a significant negative correlation with the increased ReHo in the right LG (*r* = −0.436, *p* = 0.048, Figure 7G) and decreased TG (*r* = −0.44, *p* = 0.048, Figure 7H).

## Discussion

In this study, rsfMRI was used to investigate whether IER in obese individuals trying to lose weight is accompanied with changes in spontaneous neural activity. The results revealed that IER significantly improved anthropometric measurements, biochemical indicators, and adipokine levels. The IER intervention in weight loss was associated with a significant increase in ReHo in the bilateral LG, left calcarine, and left PCG and a significant decrease in the right MTG and right cerebellum (VIII). Follow-up analyses showed that the increase in ReHo values in the right LG had a significant positive correlation with a reduction in TFEQ-disinhibition and a significant negative correlation with an increase in TFEQ-cognitive control. Furthermore, the increase in ReHo values in the left calcarine had a significant positive correlation with the reduction in TFEQ-disinhibition. Altogether, these results supported our hypothesis that the IER intervention induced changes in ReHo, and these improvements were associated with changes in anthropometric measurements, biochemical indicators, and adipokines.

Previous studies have indicated that IER is a feasible, effective, and acceptable intervention for treating overweight and obese adults (Harris et al., 2018; Jebeile et al., 2019; Wei et al., 2022). The common types of IER diets are alternate-day fasting (ADF), 5:2 diet, week-on-week-off diet, and time-restricted feeding (Wei et al., 2022). For adult patients with obesity, this study adopted the ADF method most commonly used in previous studies (Wei et al., 2017; Zeng et al., 2021). However, the current study improved it by designing two phases: HCFP and LCFP. In the HCFP, to allow obese patients to adapt to daily calorie restriction, we designed four stages to gradually reduce the calorie intake of the subjects. Furthermore, the anthropometric and biochemical indicators measured in previous IER studies were not thorough (Wei et al., 2022); therefore, this study measured comprehensive indicators. The present study found decreased BW, BMI, WC, BF, PBF, SM, SBP, and DBP in individuals with obesity after 2 months of the IER intervention in the SWLG, which confirmed previous findings in the literature. Moreover, the IER intervention results for some blood biochemical indicators, including FPG, TC, TG, HDL, LDL, AST, ALT, gHbA1c, ALP, and UA, showed significant improvements across the four time points. Few previous studies have evaluated eating behaviors at different stages of IER interventions in obese patients (Sundfør et al., 2019). In this study, we used the TFEQ scale to evaluate dynamic changes in the dysfunctional eating behaviors of obese patients during the IER intervention. The results showed increased TFEQ-cognitive control and decreased TFEQ-disinhibition after the IER intervention, indicating that individuals with obesity exhibited increased conscious dietary restraint and control over eating. Furthermore, previous studies showed that increasing and maintaining cognitive restraint can facilitate successful weight maintenance (James et al., 2018; Sundfør et al., 2019). Taken together, these results confirmed the effectiveness of the IER method used in this study for weight loss.

However, whether the IER intervention is accompanied with the reshaping of brain function in obesity is currently unclear. Here, we found that ReHo of the bilateral LG and left calcarine increased after 2 months of the IER intervention. LG and calcarine belong to the visual attention network (VAN), and they are mainly involved in the processing of attention and visual information for food cues (Dietrich et al., 2016; Wang et al., 2019; Stopyra et al., 2021). Previous research indicated that abnormalities in VAN are associated with impaired concentration (Wang et al., 2019). Abnormal structure and function of the LG and calcarine have been revealed in obese patients in many studies (Pannacciulli et al., 2006; Cornier et al., 2007; Dong et al., 2014; Dietrich et al., 2016; Zhang et al., 2016; Luo et al., 2018; Stopyra et al., 2021; Yang et al., 2021). For obese individuals, neuroimaging research has uncovered reduced gray matter (GM) and white matter (WM) densities in the LG, which exhibit a negative correlation with BMI (Zhang et al., 2016). Yang et al. observed that the bilateral lingual (which has been linked to the reward value of food) presents increased activity when viewing high-calorie foods in obese individuals compared with that in lean individuals (Yang et al., 2021). The FC between the left amygdala and left LG is nearly positively related to BMI (>30 kg/m^2^) during the regulation of food cravings (Dietrich et al., 2016). Their interplay may contribute to differences in neural craving regulation (Dietrich et al., 2016).

With regard to calcarine, increased GM has been observed in obese individuals compared with lean subjects (Pannacciulli et al., 2006). Moreover, overweight individuals display increased voxel-mirrored homotopic connectivity in the calcarine compared with lean subjects (Luo et al., 2018). Several studies have found that stimulus category and body weight affect the FC of the calcarine between foods with high and low hedonic values (Cornier et al., 2007; Luo et al., 2018). Using the same method utilized in this study, a decreased ReHo of the LG and calcarine was found in non-restrained eaters relative to restrained eaters (Dong et al., 2014). Meanwhile, significant associations have been observed between post-surgery reduction in BMI and increased fractional ALFF signal (similar to ReHo in reflecting changes in the local variability of the signal) in LG (Zeighami et al., 2021). In the current study, the increase in ReHo values in the right LG and left calcarine also showed a significant positive correlation with a reduction in TFEQ-disinhibition and a significant negative correlation with an increase in TFEQ-cognitive control. These findings may indicate that the IER intervention can recover the disrupted ReHo of the LG and calcarine and exert a similar effect on the brain to that exerted by bariatric surgery. We speculated that the brains of obese persons pay particular attention to external food stimuli and communicate closely with the prefrontal regions associated with self-control after the IER intervention, thereby reducing the response of the reward system to food (Neseliler et al., 2019).

The PCG is part of the motor sensory network (MSN), and it is involved in mechanosensation and cognitive integration of sensory stimulation with emotions, memory, and the body’s internal state (Beckmann et al., 2005; Aviram-Friedman et al., 2018). The structure and function of PCG are disrupted in obesity (Cornier et al., 2007; Zhang et al., 2016; Aviram-Friedman et al., 2018). Compared with normal-weight individuals, obese individuals have significantly lower GM density in the left PCG, which is negatively associated with BMI (Cornier et al., 2007; Zhang et al., 2016). Notably, the GM density of the left PCG increased after bariatric surgery in obese individuals (Cornier et al., 2007). fMRI studies have demonstrated the great response of the PCG to palatable high-calorie drinks and visual food stimuli in adolescents with obesity, indicating the elevated responsivity of the somatosensory region to food and drinks (Aviram-Friedman et al., 2018). In this study, we observed increased ReHo values in the left PCG. Furthermore, the IER intervention resulted in a significant reduction in TFEQ-disinhibition and a significant increase in TFEQ-cognitive control, which indicated that obese individuals had enhanced self-control of their eating behavior. Therefore, enhanced cognitive control may reduce the neural activation of the PCG when stimulated by food, which in turn decreased eating behaviors to promote weight loss. These results suggested that the PCG may be a downstream output interface in reward processing, and the reward–sensory circuit plays a crucial role in external eating behaviors (Zhang et al., 2021).

The MTG is an important hub of the default mode network (DMN), which is related to the semantic representation of objects, working memory retrieval, and cognitive processes (Squire et al., 2004; Agarwal et al., 2021; Zhao et al., 2022). A meta-analysis confirmed consistent GM reductions in the MTG in obese individuals (Herrmann et al., 2019). Compared with lean individuals, obese individuals have altered FC strength in the DMN and temporal lobe network, which is associated with food regulation (Kullmann et al., 2012). In particular, the left MTG and DLPFC (implicated in dietary self-control) have negative FC (Moreno-Lopez et al., 2016; Neseliler et al., 2019). In a recent study, Zhao et al. found that the FC between the left MTG and the right angular gyrus is significantly correlated with BMI in normal-weight undergraduates, but no significant correlation with obesity exists (Zhao et al., 2022). They reported that functional changes in the MTG in obese undergraduates may be crucial for providing imaging-based biomarkers for intervention and therapy of obesity. fMRI studies have also shown that MTG activation during an episodic memory task is modulated by weight loss in overweight adults (Boraxbekk et al., 2015), and MTG responses to food stimuli predict future weight variability, which is a risk factor for obesity in adolescents (Winter et al., 2017). These results indicate that the MTG, which is critical for the regulation and maintenance of body weight, is disrupted in terms of structure, function, and interaction with other brain regions or networks. In this study, repeated-measures ANOVA indicated a significant decrease in ReHo in the right MTG after the IER intervention. The MTG has been found to increase neural activation in obese individuals exposed to food stimuli or favorite-food cues compared with normal-weight individuals (Jastreboff et al., 2013; Zhao et al., 2017). Therefore, decreased ReHo in the right MTG may reduce the semantic expression of food stimuli, which in turn reduces eating behavior.

After the IER intervention, we also found decreased ReHo in the right cerebellum (VIII), which is related to higher-order functions, including reward-based learning, attention, appetite control, emotion, and executive functions (Zhu and Wang, 2008; Lugo-Candelas et al., 2020). Obesity-related cerebellar structural and functional disturbances have been reported in many studies (Kurth et al., 2013; Contreras-Rodríguez et al., 2017; Prehn et al., 2017; Gracia-Marco et al., 2020; Lugo-Candelas et al., 2020; Liu et al., 2022; Yang et al., 2023). Their results revealed significant negative correlations between BMI, WC, and intra-abdominal adiposity and GM volume in the cerebellum (Kurth et al., 2013; Lugo-Candelas et al., 2020). Meanwhile, the WM volume of cerebellum VIII is positively associated with BMI and lean mass index (Gracia-Marco et al., 2020). Yang et al. indicated that increased structural covariances of cerebellum VIII with the perigenual anterior cingulate cortex are associated with future weight gain, which may guide the development of effective prevention and treatment interventions for obesity (Yang et al., 2023). Notably, a previous study confirmed caloric restriction-induced weight loss, which in turn reduces GM in the right cerebellum (Prehn et al., 2017). Furthermore, the decreased FC between the hypothalamus (regulating appetite and energy homeostasis) and the cerebellum, which may predict a high percentage of 3-month weight loss, indicates that the cerebellum may play a role in the regulation of eating behaviors (Contreras-Rodríguez et al., 2017; Lugo-Candelas et al., 2020). In addition, the prefrontal cortex–cerebellar circuit participates in the regulation of cognitive–emotional processes (Liu et al., 2022). Markedly increased ReHo has also been observed in the left cerebellum of subjects with severe obesity and meibomian gland dysfunction (Liu et al., 2022). In summary, the IER intervention may normalize the spontaneous neural activity of the cerebellum and promote interaction with the hypothalamus and prefrontal cortex to enhance the ability to control appetite and weight loss.

Studies have shown that the cerebellum can interact through hormonal and neural networks; specific networks, such as the hypothalamus and insula, are associated with food intake (Sader et al., 2022). In this study, we found significant decreases in leptin and adiponectin levels, consistent with previous IER weight loss results (Friedman and Mantzoros, 2015; Forny-Germano et al., 2019). Leptin reduction shows a positive correlation with decreases in BW, BMI, BF, and SM. Leptin is involved in the central control of food intake and energy homeostasis (Forny-Germano et al., 2019). Adiponectin has anti-diabetic, anti-inflammatory, and anti-atherogenic effects (Forny-Germano et al., 2019). Increased levels of leptin and decreased levels of adiponectin may promote the development of insulin resistance and type-2 diabetes, hypertension, atherosclerosis, other cardiovascular diseases, and some types of cancer (Forny-Germano et al., 2019). Leptin treatment in obese subjects prevents weight gain and supports weight maintenance (Rosenbaum et al., 2008). Moreover, the neural activities of the MTG and LG, which are known to be involved in mediating the executive and decision-making functions of food intake, were reversed by leptin (Rosenbaum et al., 2008) and regulated by IER in the present study. Leptin receptors are densely expressed in the cerebellum and involved in cerebellar responsiveness to food cues (Lugo-Candelas et al., 2020). Berman et al. found that a long duration of leptin replacement is associated with increased activation of food images in the ventral portion of the posterior lobe of the cerebellum (Berman et al., 2013). Altogether, our results indicated that leptin may interact with regions of the cerebellum, MTG, and LG to control eating behavior during the IER intervention. However, no correlation was found between the significantly changed leptin levels and ReHo of the brain region induced by the IER intervention, possibly because changes in leptin are closely related to changes in other brain area indicators. However, this result requires further investigation.

## Conclusion

In summary, our findings supported our hypothesis that IER reshaped ReHo in some brain regions in obese individuals who successfully lost weight after the IER intervention, accompanied with improved anthropometric measurements, biochemical indicators, and adipokines. The improved regions were mainly located in the VAN, MSN, DMN, and cerebellum, which were related to attention, food reward stimulation, cognitive processes and integration, and appetite control. These results illustrated that the IER intervention for weight loss decreased the motivational drive to eat, reduced reward responses to food cues, and repaired damaged food-related self-control processes. These findings enhance our understanding of the neurobiological basis of IER for weight loss in obesity. However, double-blind sham-controlled trials with a large sample size are needed to confirm the results of this initial study.

## Limitations

This study had several limitations that should be considered. First, we did not include a sham intervention group. We focused on changes in brain activity during the IER intervention and their relationship with obesity-related indicators. Previous studies have confirmed that IER is a feasible, effective, and acceptable intervention for treating individuals with obesity (Harris et al., 2018; Jebeile et al., 2019; Sundfør et al., 2019; Wei et al., 2022). Moreover, some studies have investigated altered ReHo in the brains of obese individuals (Dong et al., 2015; Zhang et al., 2015; Gao et al., 2018; Zeighami et al., 2021; Zhao et al., 2022). Therefore, the sham group was not included in the present study. Second, this study implemented a short-term (2 months) IER intervention for obesity. The hard part of weight loss is not losing weight, but potential weight regain often occurs after the intervention is terminated. Further follow-up studies are needed to reveal long-term changes in spontaneous neural activity that affect long-term weight loss. Finally, a larger sample than that used in this study is needed to verify the results of this initial study.

## Data availability statement

The original contributions presented in the study are included in the article/Supplementary material, further inquiries can be directed to the corresponding authors.

## Ethics statement

The studies involving human participants were reviewed and approved by Henan Provincial People’s Hospital. The patients/participants provided their written informed consent to participate in this study.

## Author contributions

ZL, XW, HG, and TX conceived the study, analyzed the data, and wrote the manuscript. JZ, ZZ, LT, BY, CZ, LW, and HL designed and performed the experiments. WW, TY, FL, HM, XZ, NM, and ZY collected the clinical samples and performed the experiments. QZ and YL conceived the study, designed the experiments, supervised the project, and wrote the manuscript. All authors contributed to the article and approved the submitted version.

## Funding

This study was supported by the National Natural Science Foundation of China (82071884), Science and Technology Project of Henan Provincial Science and Technology Department (222102310198), Young and Middle-aged Health Science and Technology Innovative Talent Cultivation Project of Henan Provincial Leading Talents (YXKC2020004), and Medical Science and Technology Research Program of Henan Province (RKX202202013).

## Conflict of interest

The authors declare that the research was conducted in the absence of any commercial or financial relationships that could be construed as a potential conflict of interest.

## Publisher’s note

All claims expressed in this article are solely those of the authors and do not necessarily represent those of their affiliated organizations, or those of the publisher, the editors and the reviewers. Any product that may be evaluated in this article, or claim that may be made by its manufacturer, is not guaranteed or endorsed by the publisher.

## Footnotes

^1^http://rfmri.org/DPABI
