# Supplementary material for: Intermittent energy restriction changes the regional homogeneity of the obese human brain
Source: Front Neurosci. 2023 Aug 3;17:1201169. doi: 10.3389/fnins.2023.1201169 (PMC10434787; doi:10.3389/fnins.2023.1201169)
Supplement: Supplementary file 1 [file Data_Sheet_1.docx]

**Supplementary Material Figure and Table**

**
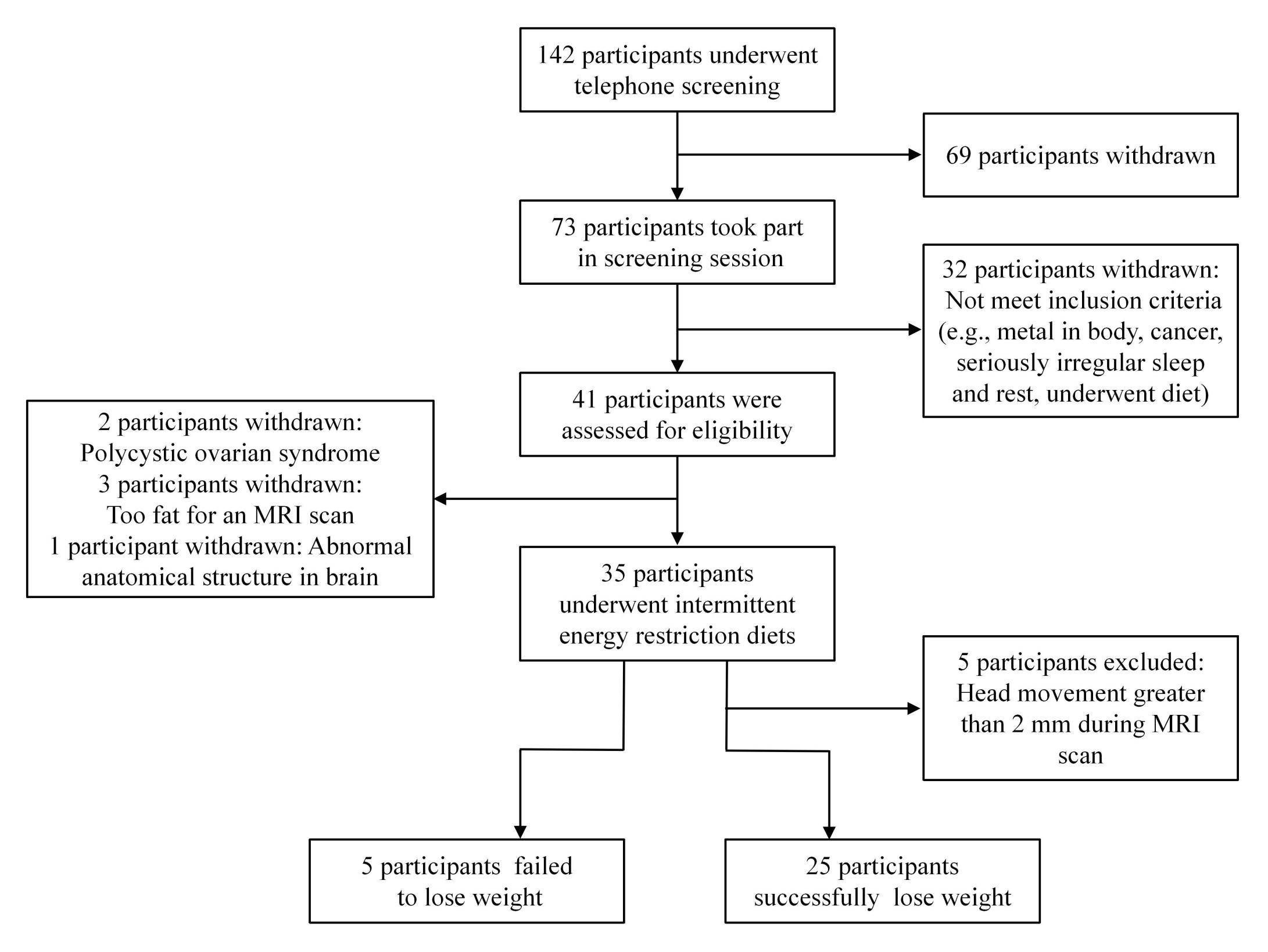
**

**Figure S1.** A schematic overview of recruitment of participants.

**Table S1**. Time effects and post-hoc tests for clinical characteristics of participants at baseline, MHCFP, EHCFP, and ELCFP during IER intervention.

| Variable | Successful Weight Loss Group (25 Subjects) | | | | Failed Weight Loss Group (5 Subjects) | | | | ANOVA for Successful Weight Loss Group | | | | | | | |
| --- | --- | --- | --- | --- | --- | --- | --- | --- | --- | --- | --- | --- | --- | --- | --- | --- |
|  | Baseline  (Mean ± SD) | MHCFP  (Mean ± SD) | EHCFP  (Mean ± SD) | ELCFP  (Mean ± SD) | Baseline  (Mean ± SD) | MHCFP  (Mean ± SD) | EHCFP  (Mean ± SD) | ELCFP  (Mean ± SD) | ANOVA  Time effect | | Post-hoc tests | | | | | |
|  |  |  |  |  |  |  |  |  |  |  | MHCFP vs. Baseline 21 | EHCFP vs. Baseline 31 | EHCFP vs. MHCFP32 | ELCFP vs. Baseline 41 | ELCFP vs. MHCFP 42 | ELCFP vs. EHCFP 43 |
|  |  |  |  |  |  |  |  |  | F | *P* value | *P* value | *P* value | *P* value | *P* value | *P* value | *P* value |
| Body weight (kg) | 97.53 ± 15.67 | 92.91 ± 15.40 | 89.92 ± 14.98 | 88.89 ± 15.61 | 93.18 ±11.66 | 91.16 ± 12.13 | 90.88 ± 11.64 | 91.28 ± 11.76 | F(3,72) = 117.54 | **＜0.001** | **＜0.001** | **＜0.001** | **＜0.001** | **＜0.001** | **＜0.001** | 0.386 |
| Body Mass Index (kg/m2) | 34.64 ± 4.05 | 32.98 ± 3.93 | 31.94 ± 3.95 | 31.52 ± 3.85 | 34.02 ± 2.87 | 33.20 ± 3.16 | 33.12 ± 2.90 | 33.24 ± 2.88 | F(3,72) = 103.78 | **＜0.001** | **＜0.001** | **＜0.001** | **＜0.001** | **＜0.001** | **＜0.001** | 0.382 |
| Waist Circumference (cm) | 108.40 ± 11.33 | 105.00 ± 11.35 | 101.59 ± 12.63 | 100.75 ± 11.61 | 107.00 ± 8.31 | 108.60 ± 4.67 | 105.6 ± 3.13 | 108.20 ± 3.72 | F(3,69) = 29.85 | **＜0.001** | **0.008** | **＜0.001** | **0.004** | **＜0.001** | **＜0.001** | 1 |
| Body Fat (kg） | 39.46 ± 9.44 | 36.40 ± 8.97 | 34.36 ± 9.17 | 32.79 ± 9.03 | 36.92 ± 7.17 | 35.70 ± 7.68 | 35.12 ± 7.35 | 35.00 ± 7.48 | F(3,72) = 126.41 | **＜0.001** | **＜0.001** | **＜0.001** | **＜0.001** | **＜0.001** | **＜0.001** | **0.002** |
| Percent of Body Fat (%) | 40.34 ± 6.11 | 39.05 ± 6.13 | 38.02 ± 6.61 | 36.66 ± 6.34 | 39.44 ± 4.09 | 38.90 ± 4.39 | 38.42 ± 4.26 | 38.10 ± 4.49 | F(3,72) = 64.07 | **＜0.001** | **＜0.001** | **＜0.001** | **0.007** | **＜0.001** | **＜0.001** | **＜0.001** |
| Skeletal Muscle (kg) | 32.55 ± 6.21 | 31.66 ± 6.13 | 31.00 ± 5.97 | 31.20 ± 6.07 | 31.48 ± 3.72 | 31.10 ± 3.59 | 31.20 ± 3.40 | 31.50 ± 3.57 | F(3,72) = 27.53 | **＜0.001** | **＜0.001** | **＜0.001** | **0.001** | **＜0.001** | 0.262 | 1 |
| Systolic Blood Pressure (mmHg) | 128 ± 15 | 122 ± 11 | 122 ± 13 | 119 ± 13 | 128.25 ± 21.69 | 125.75 ± 11.53 | 123.5 ± 13.28 | 127.5 ± 11.90 | F(3,72) = 5.635 | **0.002** | **0.039** | 0.180 | 1 | **0.001** | 0.898 | 1 |
| [Diastolic Blood Pressure](file:///E:/Youdao/Dict/8.8.0.0/resultui/html/index.html#/javascript:;) (mmHg) | 79 ± 13 | 75 ± 9 | 72 ± 10 | 73 ± 11 | 80.75 ± 13.07 | 75.75 ± 8.85 | 73.00 ± 7.44 | 79.50 ± 10.34 | F(3,72) = 4.324 | **0.007** | 0.530 | 0.095 | 0.575 | 0.074 | 1 | 1 |
| TFEQ (Disinhibition) | 22.65 ± 4.87 | 17.43 ± 5.05 | 17.04 ± 6.50 | 18.04 ± 5.59 | 17 ± 6.58 | 16.75 ± 7.27 | 19.75 ± 2.75 | 14.75 ± 5.68 | F(3,66) = 8.3757 | **＜0.001** | **0.005** | **0.001** | 1 | **0.005** | 1 | 1 |
| TFEQ (Cognitive Control) | 15.65 ± 2.90 | 19.13 ± 3.02 | 19.13 ± 3.70 | 18.08 ± 2.43 | 15.75 ± 2.50 | 16.50 ± 3.42 | 16.75 ± 2.63 | 16 ± 1.83 | F(3,66) = 9.984 | **＜0.001** | **＜0.001** | **0.003** | 1 | **0.007** | 0.722 | 1 |
| TFEQ (Hunger) | 12.17 ± 4.11 | 10.52 ±4.69 | 10.74 ± 5.26 | 12.70 ± 5.67 | 10.50 ± 5.74 | 10.00 ± 4.24 | 11.75 ± 1.26 | 10.00 ± 4.90 | F(3,66) = 1.884 | 0.141 | 0.164 | 0.782 | 1 | 1 | 0.520 | 1 |

Results are presented as mean ± SD; repeated-measures ANOVA comparing baseline, MHCFP, EHCFP, and ELCFP during IER intervention. Abbreviation: midpoint of highly-controlled fasting phase, MHCFP; endpoint of highly-controlled fasting phase, EHCFP; endpoint of low-controlled fasting phase, ELCFP; intermittent energy restriction, IER; standard deviation, SD, three factor eating questionnaire, TFEQ.

**Table S2**. Time effects and post-hoc tests for blood biochemical indicators of participants at baseline, MHCFP, EHCFP, and ELCFP during IER intervention.

| Variable | Successful Weight Loss Group (25 Subjects) | | | | Failed Weight Loss Group (5 Subjects) | | | | ANOVA for Successful Weight Loss Group | | | | | | | |
| --- | --- | --- | --- | --- | --- | --- | --- | --- | --- | --- | --- | --- | --- | --- | --- | --- |
|  | Baseline  (Mean ± SD) | MHCFP  (Mean ± SD) | EHCFP  (Mean ± SD) | ELCFP  (Mean ± SD) | Baseline  (Mean ± SD) | MHCFP  (Mean ± SD) | EHCFP  (Mean ± SD) | ELCFP  (Mean ± SD) | ANOVA  Time effect | | Post-hoc tests | | | | | |
|  |  |  |  |  |  |  |  |  |  |  | MHCFP vs. Baseline | EHCFP vs. Baseline | EHCFP vs. MHCFP | ELCFP vs. Baseline | ELCFP vs. MHCFP | ELCFP vs. EHCFP |
|  |  |  |  |  |  |  |  |  | F | *P* value | *P* value | *P* value | *P* value | *P* value | *P* value | *P* value |
| Fasting Plasma Glucose (mmol/L) | 4.79 ± 0.95 | 4.27 ± 0.44 | 4.07 ± 0.41 | 4.50±0.46 | 5.27 ± 1.17 | 4.82 ± 0.43 | 5.15 ± 0.98 | 5.38 ± 0.81 | F(3,72) = 14.455 | **＜0.001** | **0.002** | **＜0.001** | 0.342 | 0.404 | 0.098 | **＜0.001** |
| [Glycosylated](file:///E:/Youdao/Dict/8.8.0.0/resultui/html/index.html#/javascript:;) [Hemoglobin](file:///E:/Youdao/Dict/8.8.0.0/resultui/html/index.html#/javascript:;) (%) | 5.81 ± 0.66 | 5.90 ± 0.53 | 5.56 ± 0.45 | 5.22 ± 0.41 | 6.14 ± 0.55 | 6.20 ± 0.71 | 6.10 ± 0.42 | 5.78 ± 0.49 | F(3,72) = 71.239 | **＜0.001** | 0.092 | **0.005** | **＜0.001** | **＜0.001** | **＜0.001** | **＜0.001** |
| Total Cholesterol (mmol/L) | 4.89 ± 1.07 | 4.64 ± 1.08 | 4.47 ± 0.85 | 4.66 ± 0.89 | 4.78 ± 0.51 | 5.26 ± 0.59 | 5.16 ± 0.56 | 4.92 ± 0.68 | F(3,72) =3.788 | **0.022** | 0.315 | 0.053 | **0.036** | 0.631 | 1 | 0.697 |
| Triglycerides (mmol/L) | 2.09 ± 1.02 | 1.46 ± 0.40 | 1.63 ± 0.79 | 1.72 ± 0.60 | 3.67 ± 1.75 | 2.75 ± 0.55 | 3.36 ± 1.61 | 4.40 ± 2.90 | F(3,72) =3.835 | **0.013** | **0.034** | 0.096 | 1 | 1 | 0.809 | 1 |
| High-Density Lipoproteins (mmol/L) | 1.14 ± 0.17 | 1.04 ± 0.16 | 1.08 ± 0.15 | 1.11 ± 0.15 | 0.98 ± 0.08 | 0.96 ± 0.03 | 0.93 ± 0. 06 | 0.85 ± 0.08 | F(3,72) = 4.936 | **0.004** | **0.005** | 0.395 | 0.577 | 1 | 0.071 | 1 |
| Low-Density Lipoproteins (mmol/L) | 2.82 ± 0.86 | 2.75 ± 0.91 | 2.55 ± 0.72 | 2.63 ± 0.75 | 2.47 ± 0.20 | 2.93 ± 0.46 | 2.89 ± 0.52 | 2.36 ± 0.55 | F(3,72) = 3.587 | **0.018** | 1 | 0.069 | **0.04** | 0.209 | 1 | 1 |
| Aspartate Transaminase (U/L) | 32.40 ± 25.30 | 38.64 ± 34.28 | 29.68 ± 17.60 | 22.52 ± 6.99 | 32.00 ± 7.68 | 36.40 ± 13.69 | 32.20 ± 10.33 | 38.00 ± 18.06 | F(3,72) = 14.203 | **＜0.001** | 0.289 | 1 | 0.101 | **0.004** | **＜0.001** | **0.001** |
| Alanine Aminotransferase (U/L) | 51.32 ± 51.69 | 50.16 ± 40.04 | 41.00 ± 32.39 | 29.40 ± 23.74 | 51.00 ± 21.93 | 66.20 ± 31.59 | 47.00 ± 14.09 | 50.80 ± 10.43 | F(3,72) = 13.267 | **＜0.001** | 1 | 0.303 | 0.077 | **＜0.001** | **0.001** | **0.035** |
| Glutamyl Transpeptidase (U/L) | 44.84 ± 46.68 | 29.40 ± 22.06 | 23.20 ± 15.40 | 29.72 ± 20.21 | 79.80 ± 52.39 | 62.80 ± 35.84 | 55.20 ± 30.38 | 68.20 ± 45.57 | F(3,72) = 8.718 | **0.005** | **0.037** | **0.019** | **0.004** | 0.136 | 1 | **0.001** |
| Alkaline Phosphatase (U/L) | 65.68 ± 14.81 | 64.20 ± 13.39 | 60.20 ± 13.57 | 64.12 ± 13.77 | 76.00 ± 27.08 | 75.80 ± 29.86 | 73.20 ± 27.67 | 79.40 ± 27.68 | F(3,72) = 6.513 | **0.002** | 1 | **0.001** | **＜0.001** | 1 | 1 | 0.084 |
| Serum Creatinine (umol/L) | 65.40 ± 16.02 | 69.80 ± 14.89 | 66.76 ± 13.28 | 67.48 ± 22.15 | 54.40 ± 11.59 | 55.60 ± 11.84 | 55.60 ± 14.01 | 55.20 ± 11.17 | F(3,72) = 2.357 | 0.118 | **0.007** | 0.393 | 0.102 | 1 | 0.687 | 1 |
| Uric Acid (umol/L) | 411.12 ± 121.30 | 479.24 ± 131.50 | 429.68 ± 124.15 | 385.56 ± 118.30 | 347.70 ± 71.91 | 385.00 ± 41.94 | 359.80 ± 50.15 | 349.00 ± 81.69 | F(3,72) = 13.955 | **＜0.001** | **＜0.001** | 1 | 0.071 | 0.405 | **＜0.001** | 0.059 |
| Leptin (pg/ml) | 7319.06 ± 2910.17 | 3783.40 ± 1824.27 | 3794.81 ± 2090.77 | 4055.30 ± 1853.26 | 6448.37 ± 1866.21 | 4973.72 ± 2206.90 | 5248.93 ± 2316.42 | 2259.30 ± 686.00 | F(3,72) = 21.586 | **＜0.001** | **＜0.001** | **＜0.001** | 1 | **＜0.001** | 1 | 1 |
| Adiponectin (pg/ml) | 1038.75 ± 260.91 | 1364.86 ± 276.05 | 1336.54 ± 267.40 | 1336.54 ± 267.40 | 1032.16 ± 160.02 | 1209.16 ± 333.70 | 1174.11 ± 276.71 | 1267.18 ± 269.05 | F(3,72) = 15.334 | **＜0.001** | **＜0.001** | **0.001** | 1 | **＜0.001** | 1 | 0.986 |

Results are presented as mean ± SD; repeated-measures ANOVA comparing baseline, MHCFP, EHCFP, and ELCFP during IER intervention. Abbreviation: midpoint of highly-controlled fasting phase, MHCFP; endpoint of highly-controlled fasting phase, EHCFP; endpoint of low-controlled fasting phase, ELCFP; intermittent energy restriction, IER; standard deviation, SD.

**Table S3**. Specific daily calorie intake for four periods in the highly controlled fasting phase during intermittent energy restriction intervention

| **Time** | | **Lose Weight Diet** |
| --- | --- | --- |
| **period 1** | **Breakfast** | Oatmeal, stir-fried vegetables, boiled egg, almond, cherry tomatoes |
|  | **Lunch** | Multigrain rice, burnt eggplant, apple |
|  | **Dinner** | Soybean milk, winter melon with bell pepper, almond, multivitamins |
| **period 2** | **Breakfast** | Soybean milk, lotus vegetable salad, boiled egg, macadamia nut, cherry tomatoes |
|  | **Lunch** | Multigrain mantoue, tomatoes zucchini, sour milk |
|  | **Dinner** | Sesame paste, cucumber, macadamia nut, multivitamins |
| **period 3** | **Breakfast** | Cucumber, almond, dietary fiber |
|  | **Lunch** | Shredded lettuce, cherry tomatoes, almond, dietary fiber, multivitamins |
|  | **Dinner** | Oatmeal, shredded chicken, apple, dietary fiber |
| **period 4** | **Breakfast** | Sour milk, lettuce, walnut kernel, dietary fiber, multivitamins |
|  | **Lunch** | Cucumber, scrambled eggs with carrots, dietary fiber |
|  | **Dinner** | Cherry tomatoes, kiwi fruit, walnut kernel, dietary fiber |
